# Food Aspiration Induced Hypoxic Encephalopathy Leading to Status Epilepticus

**DOI:** 10.7759/cureus.26766

**Published:** 2022-07-12

**Authors:** Kenichi Oshikiri, Ryuichi Ohta, Chiaki Sano

**Affiliations:** 1 Community Medicine Management, Shimane University Faculty of Medicine, Izumo, JPN; 2 Community Care, Unnan City Hospital, Unnan, JPN

**Keywords:** rural hospital, prognosis, rosc, hypoxic encephalopathy, cardiopulmonary arrest, seizure

## Abstract

Cardiopulmonary arrest (CPA) is an emergency that can easily lead to death without appropriate life support. Even if the return of spontaneous circulation (ROSC) is achieved, survivors of sudden cardiac arrest have sustained various degrees of hypoxic encephalopathy. In Japan, rural community hospitals tend to provide care to patients with status epilepticus caused by hypoxic encephalopathy after CPA without sufficient resources. These hospitals neither have enough staff or equipment nor can they perform all the tests required to accurately estimate the prognosis. However, simple methods can be used for better estimation, including reviewing information on arrival, physical examinations, and imaging tests. Herein, we report a case of status epilepticus caused by hypoxic encephalopathy due to food aspiration in a 72-year-old man. For the sake of patients and their families, hospitals without sufficient staff or equipment should try to estimate the prognosis of patients in a manner similar to that described in this report.

## Introduction

Cardiopulmonary arrest (CPA) is an emergency that can easily lead to death in the absence of life support. CPA can have several causes such as acute coronary syndrome, arrhythmia, dyspnea, electrolyte disturbances, and autonomic nervous system activation. The prognosis of patients with CPA depends on various factors such as the duration of the circulatory arrest, the extent of resuscitation efforts, and underlying comorbidities [1]. The pathophysiology of the causal condition also influences prognosis; there are different prognostic predictors in cardiogenic and non-cardiogenic CPA [2].

Even if the return of spontaneous circulation (ROSC) is achieved, survivors of sudden cardiac arrest have variable susceptibility to hypoxic encephalopathy. The previous study shows that among patients in coma after CPA, post-anoxic myoclonus shows massive neocortical damage and has a strong influence on the decision to withdraw life support [3]. In contrast, one systematic review of three case series that examined myoclonic status epilepticus (MSE) as a prognostic factor found that it did not have sufficient predictive ability for poor outcomes [4]. Another study concluded that seizures and myoclonus per se were not significantly related to the outcomes [5]. However, the same study suggests that status epilepticus, status myoclonus, and, in particular, MSE, are predictive of poor outcomes, as judged by survival and recovery of consciousness [5]. Although the prognosis of patients with MSE after CPA is controversial, the type of seizure should be considered when predicting the prognosis of patients with recurrent episodes of seizures who have experienced hypoxic encephalopathy after CPA.

In Japan, medium-scale community hospitals (defined as hospitals with 100-499 beds) tend to provide care to patients with status epilepticus after CPA in the absence of adequate resources. These hospitals do not have sufficient staff or equipment; for example, our hospital does not own a machine for targeted temperature management in the wards, even though induced hypothermia appears to improve the outcomes of patients in a coma after resuscitation from out-of-hospital cardiac arrest [6]. Furthermore, we cannot perform all the tests required to accurately estimate the prognosis. However, there is some evidence that can be used to estimate the neurological and survival prognoses with information on arrival, physical examinations, and imaging tests [4,7,8]. Therefore, hospitals facing a situation similar to ours have a chance of providing better care and an informed consent process.

Herein, we report a case of status epilepticus caused by hypoxic encephalopathy, which was considered a sequela of respiratory failure and subsequent CPA due to choking. The patient was refractory to initial treatment with propofol and diazepam. As a result of switching to midazolam and adding levetiracetam, fosphenytoin, and valproate sodium to his medications, his symptoms became milder but did not resolve. We attempted to estimate the patient’s neurological prognosis with information on arrival, physical examinations, imaging tests, and electroencephalography (EEG) findings.

## Case presentation

A 72-year-old man presented to our hospital with a loss of consciousness after ROSC from CPA. He lost consciousness while eating his dinner. His wife called an ambulance and started bystander cardiopulmonary resuscitation (CPR). The emergency medical service confirmed CPA and performed one cycle of CPR, which led to ROSC two minutes later. The patient was then transferred to the emergency room of our hospital. During intubation, a lump of meat was found that obstructed the upper part of the vocal cord. The lump was removed, and the obstruction was resolved.

The patient’s medical history included alcohol-induced psychosis, Wernicke encephalitis, constipation, and post carotid endarterectomy. His medications included clopidogrel 75 mg/day, amlodipine 5 mg/day, benidipine hydrochloride 4 mg/day, atorvastatin 10 mg/day, limaprost alfadex 15 μg/day, mecobalamin 1,500 μg/day, folate 5 mg/day, and yokukansan 7.5 g/day (Japanese Kampo).

On arrival, the patient was unconscious (Glasgow Coma Scale {GCS} E1VTM1). Initial vital signs were as follows: body temperature, 35.6°C; blood pressure, 130/73 mmHg; and pulse, 120 beats per minute. The emergency medical service had inserted a laryngeal tube before arriving at the hospital, and the patient was immediately placed on a ventilator at the ER. Bilateral late crackles were noted on lung auscultation. In addition, heart auscultation revealed systolic murmurs, but no sound radiating to the neck was heard. Peripheral circulatory failure or edema of the extremities was not observed. A blood test at the ER revealed hypokalemia, which was considered to be the cause of dysphagia leading to choking (Table 1). 

**Table 1:** Initial laboratory data

Marker	Level	Reference range
White blood cells	13.1	3.5–9.8 × 10^3^/μL
Neutrophils	35.3	44.0%–72.0%
Lymphocytes	51.9	18.0%–59.0%
Monocytes	9.6	0.0%–12.0%
Eosinophils	2.3	0.0%–10.0%
Basophils	0.9	0.0%–3.0%
Red blood cells	3.47	4.10–5.30 × 10^6^/μL
Hemoglobin	12.4	13.5–17.6 g/dL
Hematocrit	37.8	36.0%–48.0%
Mean corpuscular volume	108.9	82.0–101.0 fL
Platelets	25.0	13.0–36.9 × 10^4^/μL
Total protein	7.0	6.6–8.1 g/dL
Albumin	4.5	3.9–4.9 g/dL
Total bilirubin	0.3	0.2–1.2 mg/dL
Aspartate aminotransferase	56	8–38 IU/L
Alanine aminotransferase	11	4–44 IU/L
Blood urea nitrogen	16.5	8–20 mg/dL
Creatinine	0.77	0.40–1.10 mg/dL
Estimated glomerular filtration rate	75.9	>60.0 mL/min/L
Serum Na^+^	142	135–147 mEq/L
Serum K^+^	2.0	3.3–4.8 mEq/L
Serum Cl^-^	95	98–108 mEq/L
TSH	8.11	0.35–4.94 μU/mL
Free T4	0.8	0.70–1.48
Vitamin B1	24	24–66 ng/mL
Vitamin B12	308	187–883 pg/mL
Folate	6.3	3.1–20.5 ng/mL
Blood gas analysis		
pH	6.9	7.35–7.45
PCO_2_	102.0	35.0–45.0 mmHg
PO_2_	252.0	75.0–100.0 mmHg
HCO_3_	19.0	20.0–26.0 mmol/L
Base excess	−17.0	−3.0–3.0 mmol/L
Urine test		
Leukocytes	3+	
Nitrite	Negative	
Protein	+/−	
Glucose	Negative	
Urobilinogen	Negative	
Bilirubin	Negative	
Ketone	Negative	
Blood	3+	
pH	6.0	5.0–7.5
Specific gravity	1.016	

**Figure 1:**
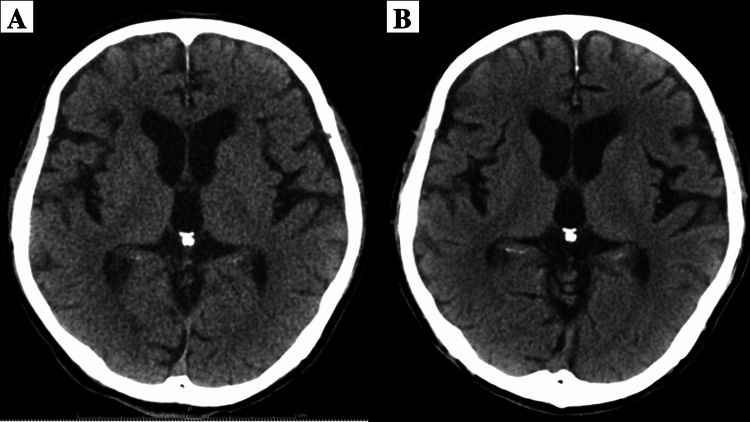
Computed tomography images of the head (A) hospital day zero and (B) one year previously, showing a mild loss of gray-white matter discrimination (GWMD) at the basal ganglia compared with a CT image taken one year earlier

Electrocardiography showed mild QT-interval prolongation. An anteroposterior chest radiograph taken at the ER showed no abnormalities. For mechanical ventilation, propofol 1.0 mL/hr and fentanyl 1.0 mL/hr were given to the patient. For hypokalemia, an intravenous drip infusion of potassium chloride was administered. In addition, the patient also received diazepam 5 mg for seizures. Computed tomography (CT) of the head after the choking episode revealed a mild loss of gray-white matter discrimination (GWMD) at the basal ganglia compared with a CT image taken one year earlier (Figure 1). Both images show cerebral atrophy due to excessive alcohol intake.

On hospital day zero (the day of hospitalization), seizures were not well controlled with diazepam 5 mg and propofol 3.0 mL/hr, which was later switched to midazolam 4.0 mL/hr. In addition, levetiracetam 1,000 mg/day and fosphenytoin sodium hydrate 750 mg were added to the treatment regimen. Norepinephrine was administered to control blood pressure.

On hospital day one, vitamin B was started for possible metabolic encephalopathy, and sodium valproate 800 mg was added for uncontrolled seizures. Body temperatures of 37.5-38.1°C were noted. On hospital day two, the fever persisted; therefore, sulbactam sodium and ampicillin sodium (SBT/ABPC) 4.5 g/day were initiated for suspected aspiration pneumonia after blood and sputum cultures were drawn. On hospital day four`, the midazolam dose was reduced to 0.1 mL/hr. Oxygen saturation decreased to 84% even after sputum aspiration. Lung auscultation revealed that the left lung was insufficiently ventilated. Floating ocular movement to the right and spasms of the diaphragm and eyelids were also noted. Spasms after the dose reduction of midazolam were suspected of unintentionally relocating the breathing tube. The dose of midazolam was increased back to 1.0 mL/hr, and the spasms subsided.

On hospital day one, intravenous vitamins B1 and 12 were started for possible metabolic encephalopathy, and sodium valproate 400 mg twice daily was added for uncontrolled seizures. On hospital day five, the levetiracetam dose was increased to 2,000 mg. On hospital day six, no seizures were noted after the withdrawal of midazolam; however, the patient remained unconscious. On hospital day seven, SBT/ABPC 4.5 g/day was switched to tazobactam/piperacillin hydrate (TAZ/PIPC) 13.5 g/day due to a body temperature of 38.5°C, which suggested ventilator-associated pneumonia. On hospital day 10, a tracheostomy was performed because it was unlikely that the patient would experience future extubation. On hospital day 11, the light reflex was intact in both pupils. The patient did not respond to his name and showed the doll’s eye phenomenon, blinks, and chewing movements. Increased biceps tendon reflex was observed. On hospital day 14, the TAZ/PIPC was discontinued.

**Figure 2:**
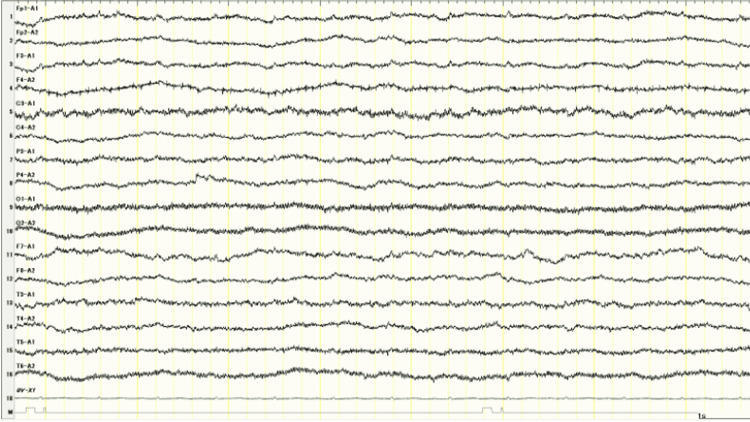
Electroencephalogram on hospital day 16

On hospital day 16, the ventilator was replaced with a heat and moisture exchanger without subsequent respiratory failure. Magnetic resonance imaging (MRI) of the head revealed areas of high signal intensity along the cerebral cortex due to hypoxic encephalopathy on diffusion-weighted images (DWI). EEG indicated low electrical activity in the brain with low amplitude, although the criterion of electrocerebral inactivity (less than 2 μV) was not met (Figure 2).

On hospital day 17, a gastrostomy was performed. On hospital day 18, the patient showed mydriasis, the doll’s eye phenomenon, and light reflex in both pupils. Normal Achilles and patellar tendon reflexes were also observed. A painful stimulus resulted in no movement of the upper extremities but an escape reaction of the lower extremities. The GCS was scored as E1VTM4. On hospital day 19, the abdomen was distended. His vital signs were as follows: body temperature, 36.9°C; blood pressure, 133/81 mmHg; pulse, 120 beats per minute; respiratory rate 24 breaths per minute; percutaneous oxygen (SpO2), 98%. CT of the abdomen revealed findings that were consistent with bowel obstruction. Gram staining of the sputum revealed gram-negative rods, cocci, and gram-positive rods. Sepsis was suspected and cefmetazole 2 g was administered. The patient was then placed on a ventilator.

On hospital day 19, the patient was unconscious and was on levetiracetam 2,000 mg and valproate 800 mg, and transferred to a nursing home.

## Discussion

In this case, the patient experienced status epilepticus caused by hypoxic encephalopathy due to food aspiration. Although medium-scale regional hospitals tend to lack sufficient resources, it is necessary to estimate the prognosis of such patients for better care and implement an informed consent process. This case shows the possibility of estimating prognosis using simple methods such as reviewing information on arrival, physical examinations, imaging tests, and EEG.

The survival rate of patients after ROSC depends on multiple clinical factors. Based on previous studies, the survival rate one month after ROSC also depends on various factors, such as age, place where the arrest occurred, bystander CPR, and the interval between the call for and arrival of the ambulance [9]. In this case, bystander CPR was performed by the patient’s wife, and an interval of seven min between the call for and arrival of the ambulance were favorable prognostic factors. In contrast, arrest at home was a poor prognostic factor.

Clinical and imaging tests may be useful for estimating and diagnosing patients with unconsciousness. In this case, CT demonstrated a mild loss of GWMD at the basal ganglia (Figure 3), which is considered a reliable early CT sign of brain hypoxia. The loss of the GWMD sign is reported to be 100% sensitive to predicting the fatal outcome [8]; however, it may depend on the severity of the loss. MRI of the head on hospital day 16 showed high signal intensity areas along the cerebral cortex, consistent with the typical image of hypoxic encephalopathy on DWI [10]. These images should be considered poor prognostic factors.

The neurological prognosis of hypoxic encephalopathy can be assessed using EEG. Generally, Hockaday’s scale is used to assess the severity of hypoxic encephalopathy based on EEG findings. Burst suppression or a flat encephalogram, defined as extremely abnormal findings on the scale, appears to predict poor neurological outcomes; however, it is difficult to estimate the prognosis of patients with low amplitudes above 2 μV. In addition, the superiority of EEG to physical examinations diminishes after day one and when M ≤ 3 is used [11]. Therefore, it is possible and rational for medium-scale regional hospitals to assess neurological prognosis based on physical examinations after day 1. This case demonstrated an escape reaction of the lower extremities to noxious stimuli on hospital day 18, which was a favorable prognostic factor; however, we cannot ignore the fact that the patient did not move the upper extremities, even in response to painful stimuli.

Symptomatic myoclonus is classified into two types: Lance-Adams syndrome and MSE in comatose patients after CPA. The former has a better prognostic value of myoclonus status when post-ROSC patients try to move their extremities consciously; however, the latter is a poor prognostic factor reported to lead to death in 89% of patients [12]. The patient experienced recurrent seizures clinically considered MSE during the first several days, although EEG was not performed before hospital day 16. We have to say that patients with MSE after hypoxic encephalopathy have a poor prognosis.

It is not easy for medium-scale regional hospitals to estimate the prognosis of patients with status epilepticus caused by hypoxic encephalopathy from food aspiration, but there are simple methods that can be used for a better estimate, as described above. This case report had some limitations. First, we were unable to perform an EEG on hospital day one because of insufficient resources [11]. Second, the patient's prognosis was not quantified. Obtaining informed consent from the patient’s family may have been easier if his prognosis was associated with a quantified probability.

## Conclusions

We report a case of status epilepticus caused by hypoxic encephalopathy due to food aspiration. Overall, it is concluded that this case has a poor neurological and survival prognosis. For the sake of patients and their families, hospitals without sufficient staff or equipment should try to estimate the prognosis of patients, similar to the manner described in this report, with information on arrival, physical examinations, imaging tests, and EEG.

## References

[1] Berek K, Jeschow M, Aichner F (1997). The prognostication of cerebral hypoxia after out-of-hospital cardiac arrest in adults. Eur Neurol.

[2] Hayakawa K, Tasaki O, Hamasaki T (2011). Prognostic indicators and outcome prediction model for patients with return of spontaneous circulation from cardiopulmonary arrest: the Utstein Osaka Project. Resuscitation.

[3] Wijdicks EF, Parisi JE, Sharbrough FW (1994). Prognostic value of myoclonus status in comatose survivors of cardiac arrest. Ann Neurol.

[4] Booth CM, Boone RH, Tomlinson G, Detsky AS (2004). Is this patient dead, vegetative, or severely neurologically impaired? Assessing outcome for comatose survivors of cardiac arrest. JAMA.

[5] Krumholz A, Stern BJ, Weiss HD (1988). Outcome from coma after cardiopulmonary resuscitation: relation to seizures and myoclonus. Neurology.

[6] Bernard SA, Gray TW, Buist MD, Jones BM, Silvester W, Gutteridge G, Smith K (2002). Treatment of comatose survivors of out-of-hospital cardiac arrest with induced hypothermia. N Engl J Med.

[7] Nadkarni VM, Larkin GL, Peberdy MA (2006). First documented rhythm and clinical outcome from in-hospital cardiac arrest among children and adults. JAMA.

[8] Inamasu J, Miyatake S, Nakatsukasa M, Koh H, Yagami T (2011). Loss of gray-white matter discrimination as an early CT sign of brain ischemia/hypoxia in victims of asphyxial cardiac arrest. Emerg Radiol.

[9] Herlitz J, Engdahl J, Svensson L, Young M, Angquist KA, Holmberg S (2004). Can we define patients with no chance of survival after out-of-hospital cardiac arrest?. Heart.

[10] Pai V, Sitoh YY, Purohit B (2020). Gyriform restricted diffusion in adults: looking beyond thrombo-occlusions. Insights Imaging.

[11] Lee YC, Phan TG, Jolley DJ, Castley HC, Ingram DA, Reutens DC (2010). Accuracy of clinical signs, SEP, and EEG in predicting outcome of hypoxic coma: a meta-analysis. Neurology.

[12] Hui AC, Cheng C, Lam A, Mok V, Joynt GM (2005). Prognosis following postanoxic myoclonus status epilepticus. Eur Neurol.

